# Chondroprotective Properties of Human-Enriched Serum Following Polyphenol Extract Absorption: Results from an Exploratory Clinical Trial

**DOI:** 10.3390/nu11123071

**Published:** 2019-12-16

**Authors:** Fabien Wauquier, Elsa Mevel, Stephanie Krisa, Tristan Richard, Josep Valls, Ruth Hornedo-Ortega, Henri Granel, Line Boutin-Wittrant, Nelly Urban, Juliette Berger, Stéphane Descamps, Jérôme Guicheux, Claire S. Vinatier, Laurent Beck, Nathalie Meunier, Adeline Blot, Yohann Wittrant

**Affiliations:** 1 Clermont Auvergne University, INRA, UNH, 63000 Clermont-Ferrand, France; Fabien_Wauquier@gmx.fr (F.W.); henrigranel@gmail.com (H.G.); line.wittrant@uca.fr (L.B.-W.); 2 Inserm, UMR 1229, RMeS, Regenerative Medicine and Skeleton, Université de Nantes, ONIRIS, F-44042 Nantes, France; elsa.mevel@hotmail.fr (E.M.); jerome.guicheux@inserm.fr (J.G.); Claire.Vinatier@univ-nantes.fr (C.S.V.); laurent.beck@inserm.fr (L.B.); 3 UFR Odontologie, Université de Nantes, F-44042 Nantes, France; 4 UR Oenologie, Université de Bordeaux, ISVV, EA 4577, USC 1366 INRA, IPB4, F-33140 Villenave d’Ornon, France; stephanie.krisa@u-bordeaux.fr (S.K.); tristan.richard@u-bordeaux.fr (T.R.); Josep.Valls-Fonayet@U-Bordeaux.Fr (J.V.); rhornedo@us.es (R.H.-O.); 5 INRAE, UMR 1019, UNH, 63122 Saint-Genès Champanelle, France; 6 Grap’sud/Inosud, 120 chemin de la regor, 30360 Cruviers-Lascours, France; NUrban@grapsud.com; 7 CRB Auvergne, Hématologie Biologique, Equipe d’Accueil 7453 CHELTER, CHU Estaing, 1 place Lucie et Raymond Aubrac, F-63003 Clermont-Ferrand, France; jberger@chu-clermontferrand.fr; 8 Orthopedics department, University Hospital Clermont-Ferrand, F-63003 Clermont-Ferrand, France; s_descamps@chu-clermontferrand.fr; 9 Rhumatology department, CHU Nantes, PHU4 OTONN, F-44042 Nantes, France; 10 CHU Clermont-Ferrand, Centre de Recherche en Nutrition Humaine Auvergne, 58 rue Montalembert, F-63000 Clermont-Ferrand, France; nmeunier@chu-clermontferrand.fr (N.M.); ablot@chu-clermontferrand.fr (A.B.)

**Keywords:** micronutrients, osteoarthritis (OA), clinical trials, hydroxytyrosol, procyanidins, cell biology

## Abstract

Polyphenols are widely acknowledged for their health benefits, especially for the prevention of inflammatory and age-related diseases. We previously demonstrated that hydroxytyrosol (HT) and procyanidins (PCy), alone or in combination, drive preventive anti-osteoathritic effects in vivo. However, the lack of sufficient clinical evidences on the relationship between dietary phytochemicals and osteoarthritis remains. In this light, we investigated in humans the potential osteoarticular benefit of a grapeseed and olive extract (OPCO) characterized for its hydroxytyrosol (HT) and procyanidins (PCy) content. We first validated, in vitro, the anti-inflammatory and chondroprotective properties of the extract on primary cultured human articular chondrocytes stimulated by interleukin-1 beta (IL-1 β). The sparing effect involved a molecular mechanism dependent on the nuclear transcription factor-kappa B (NF-κB) pathway. To confirm the clinical relevance of such a nutritional strategy, we designed an innovative clinical approach taking into account the metabolites that are formed during the digestion process and that appear in circulation after the ingestion of the OPCO extract. Blood samples from volunteers were collected following ingestion, absorption, and metabolization of the extract and then were processed and applied on human primary chondrocyte cultures. This original ex vivo methodology confirmed at a clinical level the chondroprotective properties previously observed in vitro and in vivo.

## 1. Introduction

Osteoarthritis (OA) is a common inflammatory joint disease with a strong socioeconomic impact and a growing prevalence among the aging population [1,2]. From a pathophysiological point of view, OA is characterized by progressive cartilage loss, aberrant subchondral bone remodeling, osteophyte formation, and joint tissue inflammation [3,4]. This vicious cycle of inflammation and cartilage degradation is mainly driven by interleukin-1 beta (IL-1 β). IL-1 β stimulates the synthesis of nitric oxide (NO), prostaglandin E2 (PGE2), and matrix metalloproteinases (MMPs) [5,6]. Among these proteases, MMP-13 is overexpressed in OA and mediates cartilage type II collagen and aggrecan breakdown [7] feeding the amplification loop of inflammation.

Despite recent data further depicting OA mechanisms, OA management and treatments’ efficacy remain debated. Pharmacological approaches only alleviate inflammation and pain but do not slow down, stop, or reverse the progression of the cartilage degradation [8]. Moreover, most of these drug-based approaches are associated with side effects, including bleeding, ulceration, edema, or kidney failure. In this context, alternative strategies able to blunt the progression of the disease are required. The literature has recently highlighted the potential of nutritional compounds to target osteoarticular dysfunctions such as rheumatoid arthritis [9,10] or osteoarthritis [11,12,13,14,15,16]. In this light, we investigated in humans the potential osteoarticular benefit of a grapeseed and olive extract (OPCO) characterized for its hydroxytyrosol (HT) and procyanidins (PCy) content.

Hydroxytyrosol (HT) is a bioactive phenolic compound mainly found in olive fruit and oil. HT is known for its powerful antioxidant and anti-inflammatory properties including inhibition of PGE2 and NO production pathways [17,18]. Procyanidins (PCy) are active polyphenols found in many plants such as grape, pine bark, cocoa, and raspberry. PCy from grape seed extract has been reported to alleviate inflammation, to reduce NO and PGE2 both in vitro and in vivo, [19] and to blunt cartilage degradation during OA [17,20]. In a recently published paper, we demonstrated that hydroxytyrosol (HT) and procyanidins (PCy) used alone or in combination prevent post-traumatic osteoarthritis damages in both a destabilization of the medial meniscus (DMM) mouse model and an anterior cruciate ligament transection (ACLT) rabbit model and exhibit anti-IL-1 β activities in vitro and ex vivo in rabbit articular chondrocytes (RAC) cultures. However, the relationship between dietary phytochemicals and osteoarthritis at a clinical level remains unclear due to the lack of sufficient evidence [21].

Actually, despite this large body of in vitro data, “real life” cells within the body never interact with native nutrients. At a body level, cells within tissues deal with nutrient-derived metabolites [22] following oral administration. Along the digestive track, polyphenols undergo several chemical modifications before reaching the bloodstream [23,24]. Consistent with our previous in vitro and in vivo data regarding HT and PCy influence on OA onset, we investigated the relevance of such a nutritional strategy in humans. In this purpose, we designed a pioneering approach considering metabolism at the whole-body level to decipher whether and how different plant extracts enriched in HT and PCy may clinically benefit OA management.

## 2. Materials and Methods

### 2.1. Ethics Clinical Trial

The investigations were carried out following the rules of the Declaration of Helsinki of 1975 (https://www.wma.net/what-we-do/medical-ethics/declaration-of-helsinki/) revised in 2013. The human study was approved by the French Ethical Committee (Comité de Protection des Personnes (CPP17048/N° IDRCB: 2017-A02543-50) of Saint-Germain-en-Laye—Ile de France XI). No safety signals were reported following extract ingestion. The volunteers were informed on the objectives of the present study and on the potential risks of ingestion of polyphenols, such as diarrhea and abdominal pain.

### 2.2. Human Study Design 

A pool of 20 healthy men (20 to 30 years old; average: 24 years old; average BMI of 23.35 kg/m^2^; >50 kg; without treatment; and no distinction on ethnicity) volunteered for this study. They were tested for normal blood formulation and for renal and liver functions (aspartate aminotransferase (AST), alanine aminotransferase (ALT), gamma-glutamyltransferase (GGT), urea, and creatinine). Blood samples of all participants were obtained and collected in Vacutainer Ethylenediaminetetraacetic acid (EDTA)-containing tubes and serum-separating tubes for peripheral blood mononuclear cells (PBMCs) isolation and serum separation, respectively. Biological samples were prepared, aliquoted, and stored at the Centre de Ressources Biologiques (CRB)-Auvergne, a specialized laboratory that guarantees the quality of samples and compliance with regulatory and ethical obligations (certification according to the French standard NF S 96 900). The first step of the study aimed at determining the polyphenol absorption peak. Five healthy volunteers who fasted for 12 h were given at once 3.2 g of a grapeseed and olive extract (OPCO) characterized for its hydroxytyrosol (HT) and procyanidins (PCy) content. The dose was set according to validated preclinical data [23,24]. The OPCO extract was given under the form of 8 caps (400 mg each) with 200 mL of water. OPCO extract is a combination of grapeseed and olive extract with a guaranteed content in hydroxytyrosol (6%) and procyanidines (15%) and with a 90% total polyphenol content. Approximately 10 mL of venous blood was collected from the cubital vein, before and every 20 min after the ingestion for a total period of 240 min. Serum was prepared from venous blood samples and stored at −80 °C until analysis. Polyphenol absorption profiles were quantified by ultra-high performance liquid chromatography (UPLC) coupled mass spectrometry. Once the absorption peak was determined, volunteers were called back for the collection of the enriched serum fraction. Twenty healthy volunteers who fasted for 12 h were given 3.2 g of OPCO extract under the form of 8 caps with 200 mL of water. Approximately 110 mL of venous blood was drawn from the cubital vein before the ingestion for the collection of a naïve serum. Then, at the maximum absorption peak, 100 mL of blood was drawn for enriched serum collection. Serum was stored at −80 °C until analysis.

### 2.3. Phenolic Compounds Extraction from Serum

Sera (2.5 mL) from subjects fed as described above were mixed with 7.5 mL of methanol 100% for 2 min and ultrasonicated for 5 min. The mixture was centrifuged at 20,000 g for 10 min. The supernatant was collected and evaporated to dryness using a SpeedVac Concentrator (Thermo Fisher Scientific, Illkirch, France). The dried extract was reconstituted in 200 µL of methanol/water (50:50 *v*/*v*). After centrifugation (20,000 g for 10 min), the supernatant was filtered through a polytetrafluoroethylene (PTFE) 0.22-µm filter (Millipore Corporation, Molsheim, France) and stored at −20 °C until use for ultra-high performance liquid chromatography-mass spectrometry (UPLC-MS) analysis.

### 2.4. Ultra-High Performance Liquid Chromatography-Mass Spectrometry (UPLC-MS)

Phenolic compound analyses were carried out using a 1290 Infinity UPLC (Agilent Technologies, Les Ulis, France). The UPLC system was coupled to an Esquire 3000 plus mass spectrometer from Bruker Daltonics (Wissembourg, France). Five µL was injected into a column Zorbax SB-C18 (2.1 × 100 mm, 1.8 µm) (Agilent Technologies, Les Ulis, France). Two different solvents were used as a mobile phase: solvent A (water/formic acid 99.9:0.1, *v*/*v*) and solvent B (acetonitrile/formic acid 99.9:0.1, *v*/*v*), at a flow rate of 0.4 mL/min and a gradient as follows in solvent A: 0 min 1% B, 0.4 min 1% B, 2 min 10% B, 6 min 35% B, 7 min 50% B, 8.8 min 70% B, 10.8 min 92% B, 11 min 100% B, 12 min 100% B, 12.2 min 1% B, and 15.2 min 1% B. The MS/MS parameters were set as follows: negative mode; capillary tension +4000 V; nebulizer 40 psi; dry gas 10 L/min; dry temperature 365 °C; and scan range m/z 100 to 1400. Data were processed using HyStar 3.2 software (Bruker Daltonics, Wissembourg, France).

### 2.5. OPC, PHO, and OPCO Solutions for In Vitro Experiments

Extracts were provided by Grap’Sud Union (Cruviers Lascours, France). They were characterized for their hydroxytyrosol, procyanidines, and total polyphenol contents as follows: (1) PHO extract (Olivex^®^, Grap’sud, Cruviers-Lascours, France), derived from olive, 6.5% of hydroxytyrosol/21% total polyphenol content; (2) OPC extract (Exgrape^®^ SEED, Grap’sud, Cruviers-Lascours, France), derived from grape seed, 30% of procyanidines/90% total polyphenol content; and (3) OPCO extract (Oleogrape^®^ SEED, Grap’sud, Cruviers-Lascours, France), derived from grapeseed and olive, 6.5% of hydroxytyrosol, 30% of procyanidines/90% total polyphenol content. Extracts were dissolved in Dulbecco’s Modified Eagle Medium high glucose (DMEM) (Thermo Fisher Scientific, Illkirch, France) to obtain a stock solution at 1 mg/mL. Solutions were sterilized by filtration through 0.22-µm membranes.

### 2.6. Cell Culture

Human articular chondrocytes (HACs) were harvested from tibial plateau and femoral condyles following knee replacement surgery and isolated as previously described [25]. Only intact cartilage areas were kept and processed for chondrocytes isolation. Briefly, cartilage was sliced and chips were successively digested at 37 °C with 0.05% type IV-S hyaluronidase (750–3000 units/mg) (Sigma-Aldrich, Lyon, France) in Hank’s Balanced Sodium Salt (HBSS) (Life Technologies, Villebon-Sur-Yvette, France) for 10 min and then with 0.2% trypsin (≥9000 BAEE units/mg) (Sigma-Aldrich, Lyon, France) for 15 min and with 0.2% type II collagenase (125 units/mg) (Sigma-Aldrich, Lyon, France) for 30 min. Cartilage chips were then digested overnight at 37 °C in 0.03% type II collagenase in control medium (DMEM supplemented with 10% Fetal calf serum (FCS) (Pan-Biotech, Aidenbach, Germany) and 1% penicillin/streptomycin (Life Technologies, Villebon-Sur-Yvette, France). Cells were plated at passage 1 in F225 flasks at a density of 100,000 cells/cm^2^ and maintained at 37 °C in a humidified atmosphere of 5% CO_2_ in control medium (10% FCS, 1% P/S). At confluency, cells were subcultured for in vitro and ex vivo experiments. To analyze the effects of native polyphenols or their serum metabolites, cells were preincubated for 24 h in DMEM in the presence of native OPC, PHO, or OPCO extracts at a concentration of 10 µg/mL (10% FCS and 1% P/S) or in the presence of 10% of human-enriched serum according to the Clinic’n’Cell protocol (DIRV INRA 18-00058) (1% P/S) prior an additional 24 h treatment with human recombinant IL-1 β (Millipore Corporation, Molsheim, France) at 1 ng/mL.

### 2.7. Cell Proliferation

The in vitro cell proliferation was determined using MTS Assay Kit (Abcam 197010, Paris, France). The reduction of the MTS tetrazolium compound occurs in metabolically active cells. According to the supplier’s recommendations, the generation of the formazan dye was quantified by measuring the absorbance at 490–500 nm. The ex vivo cell proliferation was determined using an XTT-based method (Cell Proliferation Kit II, Sigma-Aldrich, Lyon, France) according to the supplier’s recommendations. Optical density was measured at 450 nm.

### 2.8. NO, PGE2, and MMP-13 Quantification

Nitrate/Nitrite colorimetric assay and prostaglandin E2 Enzyme Immunoassay (EIA) kits were obtained from Cayman Chemical (Ann Arbor-MI, USA), and rabbit and human ELISA Kits for MMP-13 detection were purchased from Cloud-Clone Corp (Houston-TX, USA) and Abcam^®^ (Paris, France) respectively. The NO, PGE2, and MMP-13 levels measurements were performed according to manufacturer’s instructions. For human serum, measurements were performed in quadruplicates for each sample of the twenty volunteers.

### 2.9. Immunofluorescence Analyses

HACs were fixed in Phosphate Buffered Saline (PBS) (Life Technologies, Villebon-Sur-Yvette, France) containing 4% paraformaldehyde (PFA) (Electron Microscopy Science, Hatfield, PA, USA) and then permeabilized in PBS 0.2% Triton X-100 (Sigma-Aldrich, Lyon, France). Nonspecific binding sites were blocked and probed in PBS containing 1% Bovine Serum Albumin (BSA) (Sigma-Aldrich, Lyon, France). Rabbit anti-NF-κB p65 primary antibodies (Cell Signaling Inc., Danvers-MA, USA) were diluted 1/50 in PBS containing 1% BSA and were incubated overnight at 4 °C. Samples were incubated with Alexa Fluor^®^ 488-conjugated secondary antibody (Life Technologies, Villebon-Sur-Yvette, France) diluted 1:1000 in 1% BSA in PBS for 3 h at room temperature. Cells nuclei were stained with Hoechst 33,258 Molecular Probes^®^ (Life Technologies, Villebon-Sur-Yvette, France). HACs were counterstained with Phalloidin 594-conjugated at 1:50 in PBS for 72 h.

### 2.10. Statistical Analysis

Each experiment was performed at least in triplicate. Results are expressed as mean ± SEM (standard error of the mean). Statistical analyses were carried out using ExcelStat Pro (Microsoft, Issy-les-Moulineaux, France). One-way ANOVA followed by Tukey’s test or *T-test* were performed. Groups with significant differences (*P* < 0.05) are indicated with different letters or * versus.

## 3. Results

### 3.1. OPCO Extract Reduces IL-1ß-Induced Levels of NO, PGE2, and MMP-13 Production in Human Articular Chondrocytes (HACs)

**Figure 1:**
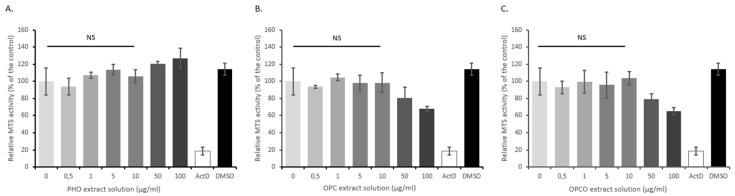
PHO, OPC, and OPCO effects on human articular chondrocytes proliferation rate. Human articular chondrocytes (HACs) were harvested from tibial plateau and femoral condyles following knee replacement surgery and isolated. Extracts were dissolved in Dulbecco’s Modified Eagle Medium high glucose (DMEM) (Thermo Fisher Scientific, Illkirch, France) to obtain a stock solution at 1 mg/mL. Solutions were sterilized by filtration through 0.22-µm membranes. Cells were preincubated with PHO (**A**), OPC (**B**), or OPCO (**C**) extract solutions at different concentrations ranging from 0.5 to 100 µg/mL for 24 h and stimulated with IL-1 β (1 ng/mL) for additional 24 h. Effects of PHO, OPC, or OPCO on MTS activity were measured. PHO, OPC, or OPCO extracts are characterized for their content in hydroxytyrosol, procyanidins or both, respectively. Concentrations below 10 µg/mL have no significant impact on cell proliferation. NS: no significant difference compared to control condition (0 µg/mL).

We first ascertained the effects of PHO, OPC, and OPCO extracts on cell viability. HACs were treated for 72 h with increasing concentrations of the different extracts (0, 0.5, 1, 5, 10, 50, and 100 µg/mL), and MTS activity was evaluated. When chondrocytes were cultured with actinomycin-D, a cell death inducer, MTS activity was significantly reduced by 80% as compared to its vehicle (DMSO –Dimethylsulfoxyde) (Figure 1). OPC and OPCO significantly decreased the MTS activity for concentration higher than 50 µg/mL in HAC. Consistently, the 10 µg/mL concentration for each extract was chosen for each extract and used for all subsequent experiments.

**Figure 2:**
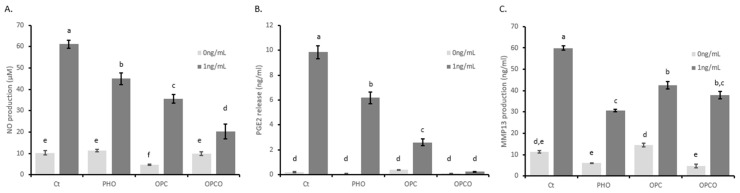
PHO, OPC, and OPCO effects on nitric oxide (NO), prostaglandin E2 (PGE2), and matrix metalloproteinase (MMP-13) production in human articular chondrocytes: Human articular chondrocytes (HACs) were harvested from tibial plateau and femoral condyles following knee replacement surgery and isolated. Extracts were dissolved in Dulbecco’s Modified Eagle Medium high glucose (DMEM) (Thermo Fisher Scientific, Illkirch, France). Solutions were sterilized by filtration through 0.22-µm membranes. Cells were preincubated with PHO, OPC, or OPCO extract solutions at 10 µg/mL for 24 h and stimulated with IL-1β (1 ng/mL) for additional 24 h. Effects of PHO, OPC, and OPCO on NO (**A**), PGE2 (**B**), and MMP13 (**C**) release in culture media were measured. PHO, OPC, or OPCO extracts are characterized for their content in hydroxytyrosol, procyanidins, or both. Groups with significant differences (*P* < 0.05) are indicated with different letters (a, b, c, d, e and f).

To decipher the in vitro effects of PHO, OPC, and OPCO extracts on molecular mechanisms underlying OA, we evaluated whether extracts may modulate the expression levels of IL-1 β-responsive genes involved in inflammation and catabolic processes in cartilage. HACs were pre-treated with PHO, OPC, or OPCO extracts for 24 h and then stimulated or not with IL-1 β for additional 24 h. Levels of NO, PGE2, and MMP-13 were measured. As expected, IL-1 β treatment increased the production of markers involved in inflammation (NO +498% and PGE2 +4820%) and catabolism (MMP13 +427%) by human articular chondrocytes (Figure 2). This production was significantly limited in the presence of polyphenols from the different extracts. OPCO extract showed a greater limitation than both PHO and OPC on inflammatory marker production (NO from +498% to +104% of the control condition; PGE2 from +4820% to +15% of the control condition) (Figure 2A,B). Actually, OPCO totally suppressed the PGE2 production triggered by IL-1 β (Figure 2B). In contrast, although OPC, PHO, and OPCO extracts reduced MMP13 production (from +427% to +169% (PHO); from +427% to +275% (OPC); and from +427% to +234% (OPCO) of the control condition), there was no major difference between these three extracts (Figure 2C).

### 3.2. OPCO Extract Decreases the IL-1 β-Mediated Activation of the NF-κB p65 Signaling Pathway

**Figure 3:**
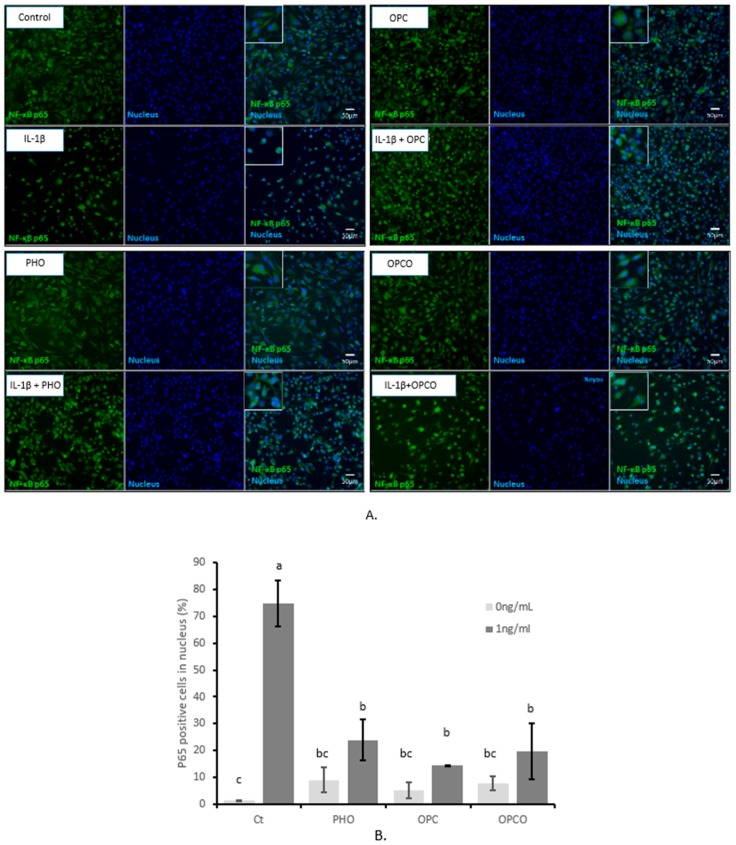
PHO, OPC, and OPCO effects on the IL-1 β-dependent nuclear transcription factor-kappa B (NF-κB) p65 translocation in human articular chondrocytes: Human articular chondrocytes (HACs) were harvested from tibial plateau and femoral condyles following knee replacement surgery and isolated. Extracts were dissolved in Dulbecco’s Modified Eagle Medium high glucose (DMEM) (Thermo Fisher Scientific, Illkirch, France). Solutions were sterilized by filtration through 0.22-µm membranes. Cells were preincubated with PHO, OPC, or OPCO extract solutions at 10 µg/mL for 24 h and stimulated with IL-1 β (1 ng/mL) for additional 24 h. Immunofluorescence assay for p65 subunit (**A**) was performed, and p65 positive cells in nucleus was counted (**B**). PHO, OPC, and OPCO extract solutions limited IL-1 β-induced p65 translocation to the nucleus. PHO, OPC, or OPCO extracts are characterized for their content in hydroxytyrosol, procyanidins, or both. Groups with significant differences (*P* < 0.05) are indicated with different letters (a, b and c).

To determine the mechanism of action, we questioned whether IL-1 β-activated signaling pathway may be modulated by the presence of the polyphenols of the extracts. NF-κB is an essential transcriptional regulator of IL-1 β-activated inflammatory and catabolic mediators including NO, PGE2, and MMP13. After 24 h of pretreatment with the different extracts at 10 µg/mL followed by an additional 24 h stimulation period with IL-1 β (1 ng/mL), the NF-κB p65 subunits were visualized by immunofluorescence assays in human articular chondrocytes (Figure 3). In untreated conditions, p65 NF-κB fragment mainly remained cytoplasmic with only a few human articular chondrocytes positive for nuclear p65 NF-κB fragment labelling (1.4%). In contrast, following IL-1 β incubation, the nuclear staining pattern for p65 NF-κB reached 74.7%, thus indicating a massive nuclear translocation. Pretreatment with PHO extract, OPC extract, or OPCO extract decreased nuclear staining of p65 NF-κB. According to fluorescence quantification, the limitations of the IL-1 β-induced nuclear translocation were −50.7%, −60.2%, and 54.8% for PHO, OPC, and OPCO respectively. Interestingly, p65 translocation was slightly but insignificantly upregulated by PHO, OPC, or OPCO extracts in the absence of IL-1 β stimulation.

### 3.3. Human-Enriched Sera Confirm the Chondroprotective Influence of Extracts

**Figure 4:**
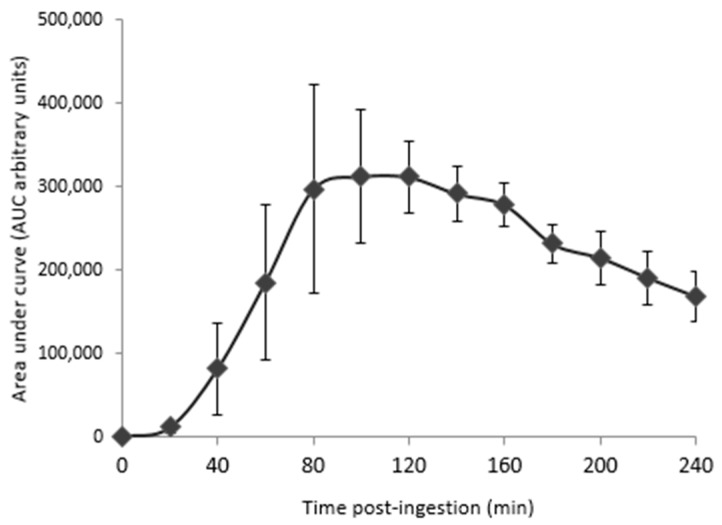
Pharmacokinetics for metabolites in the serum from peripherical blood from 0 to 240 min after the ingestion of the OPCO extract: 5 healthy volunteers who fasted for 12 h were given 3.2 g of a grapeseed and olive extract (OPCO) characterized for its hydroxytyrosol (HT) and procyanidins (PCy) content. The dose was set according to validated preclinical data. The OPCO extract was given under the form of 8 caps with 200 mL of water. Venous blood was collected from the cubital vein before and every 20 min after the ingestion for a total period of 240 min. Data are expressed as the mean values of 5 volunteers. Standard errors are depicted by vertical bars. The 100 min time point was chosen for enriched serum collection.

To get closer to a physiological context and to further investigate the anti-IL-1 β effects of OPCO extract, we validated the influence of the OPCO extract at a clinical level. Fasted volunteers received 3.2 g of OPCO extract. The digestion and absorption profile of the extract was monitored. Modulation of the polyphenol metabolite concentrations in human serum was measured by UPLC-MS. As shown in Figure 4, the cumulative area under curve (AUC) for all metabolites rapidly reached a peak at 100 min, corresponding to the maximum metabolite concentration time point. Consequently, enriched serum with OPCO metabolites was collected at 100 min post-ingestion.

**Figure 5:**
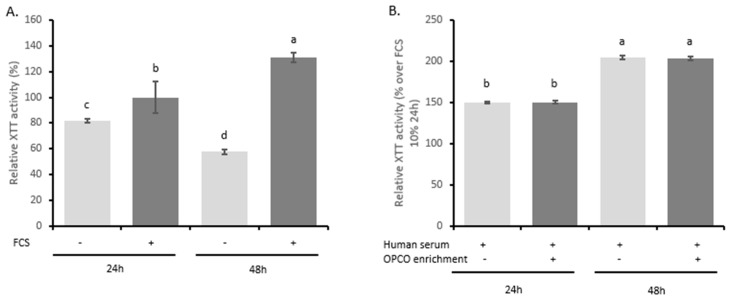
Effect of fetal calf serum and human serum enriched with OPCO metabolites on primary human articular chondrocytes proliferation: XTT activity. Human articular chondrocytes (HACs) were harvested from tibial plateau and femoral condyles following knee replacement surgery and isolated. Human primary chondrocytes were preincubated with serum from calf (**A**) or human origin (**B**) for 24 and 48 h. Primary human articular chondrocytes proliferation was measured using the XTT-based assay. Cells proliferate without any negative impact of human serum or metabolite enrichment (− absence; + presence). Groups with significant differences (*P* < 0.05) are indicated with different letters (a, b, c and d).

Then, we checked the influence of the different sera on cell proliferation and viability in primary human articular chondrocytes by measuring XTT-based activity. As expected, cell growth stopped in serum free cultures (−18% and −42% after 24 h and 48 h, respectively, compared to 24 h of FCS 10%) while cells proliferated in the presence of FCS 10% (+31% between 24 and 48 h) (Figure 5A). Naïve or enriched human serum processed according to the Clinic’n’Cell methodology (DIRV#18-0058; see the Patents section) did not exert any cytotoxic effect on cell growth as compared to regular fetal calf serum and allowed cell proliferation similar to 10% FCS treatment (+35% between 24 and 48 h) (Figure 5B) [22].

**Figure 6:**
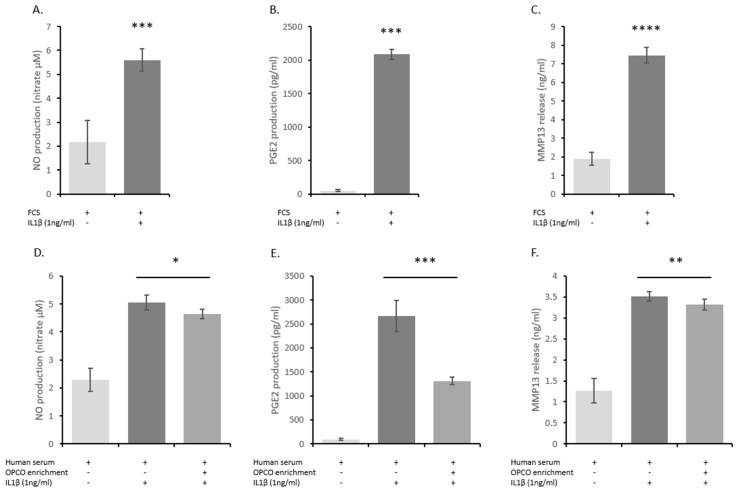
Effect of human serum enriched with OPCO metabolites on NO, PGE2, and MMP-13 production in primary human articular chondrocytes: Human articular chondrocytes (HACs) were harvested from tibial plateau and femoral condyles following knee replacement surgery and isolated. HACs were preincubated with fetal calf serum for 24 h and stimulated with IL-1 β (1 ng/mL) for additional 24 h (**A**–**C**). To analyze the effects of human serum enriched with metabolites, cells were preincubated for 24 h in DMEM in the presence of 10% of human-enriched serum according to the Clinic’n’Cell protocol (DIRV INRA 18-00058) (1% P/S) prior an additional 24 h treatment with human recombinant IL-1β (Millipore Corporation, Molsheim, France) at 1 ng/mL (**D**–**F**). NO (**A**,**D**), PGE2 (**B**,**E**), and MMP13 (**C**,**F**) release in culture media were measured. IL-1 β stimulated NO, PGE2, and MMP13 release independently of the origin of the serum (calf or human). The human serum enriched with OPCO metabolites significantly limited NO, PGE2, and MMP13 release. * (*P* < 0.05); ** (*P* < 0.01); *** (*P* < 0.001); **** (*P* < 0.0001).

To validate our cellular model and ex vivo methodology, we validated the influence of IL-1 β on cells in the presence of either FCS or human serum. As expected, IL-1β upregulated the production of NO, PGE2, and MMP13 in human primary articular chondrocytes. Upregulation occurred independently of the type of serum, FCS (Figure 6A–C) or human (Figure 6D–F), confirming the physiological relevance of both the model and the approach. Percentages of induction were similar in the presence of FCS or human serum reaching +159% and +121% for NO, +3982% and +2697% for PGE2, and +296% and +211% for MMP13, in the presence of FCS or human serum, respectively. Interestingly, as previously observed in vitro, the serum enrichment in OPCO extract metabolites significantly reduced the IL-1 β-induction of those three markers (−8.1% for NO, −50.7% for PGE2, and −5.8% for MMP13 production), validating the positive influence of OPCO on articular chondrocytes in humans.

## 4. Discussion

In this study, we demonstrated that PHO, OPC, or OPCO polyphenol extracts, characterized for their content in hydroxytyrosol and/or procyanidins, exhibit anti-IL-1 β activities in human articular chondrocytes cultures both in vitro and ex vivo using an innovative human serum-enriched approach considering their metabolism. Despite outlying discrepancies between in vitro and ex vivo data, both approaches lead to the same conclusions and greatly match with the well-acknowledged anti-inflammatory capabilities of polyphenols [9]. For instance, in an experimental collagen-induced arthritis (CIA) mice arthritis model, extra-virgin olive-oil polyphenol extract containing hydroxytyrosol was proven to exert anti-inflammatory and joint-protective effects. When given at the doses of 100 and 200 mg/kg/day, these extracts reduced the levels of proinflammatory mediators (TNF-α, IFN-γ, IL-1 β, IL-6, IL-17 A, and PGE2) [26] and decreased serum IgG1 and IgG2a, cartilage olimeric matrix protein (COMP), and metalloproteinase-3 (MMP-3) [27].

Still, there is insufficient mechanism evidences on the relationship between dietary phytochemicals and osteoarthritis, especially in humans [21]. We investigated the impact of our extracts on the NF-κB pathway as a main signaling target in NO, PGE2, and MMP13 production [28]. PHO, OPC, and OPCO drastically reduced p65 nuclear translocation induced by the presence of IL-1 β. Our results nicely fit with literature data for rheumatoid arthritis models. Hydroxytyrosol acetate (HTy-Ac) is able to lower the activation of the Janus kinase-signal transducer and activator of transcription (JAK/STAT), mitogen-activated protein kinases (MAPKs), and nuclear transcription factor-kappa B (NF-κB) pathways and thus to downregulate the arthritic process in CIA mice [26,27]. In human fibroblast-like synoviocytes (FLSs) derived from arthritic tissues, resveratrol inhibits COX-2/PGE2 expression and reduces p65 phosphorylation [29]. Others signaling pathways may be involved in polyphenol benefits but they were not investigated here. To date, resveratrol (stilbenes grape) also inhibits TNF-alpha-induced MMP-3 production in human rheumatoid arthritis fibroblast-like synoviocytes via a modulation of the PI3kinase/Akt pathway [30].

Polyphenol’s benefits on health may be potentialized by other nutrients. For instance, the combination of curcuminoid extract, hydrolyzed collagen, and green tea extract was proven to be significantly more efficient in inhibiting interleukin-1 β-stimulated matrix metalloproteinase-3 expression than curcuminoid extract alone in normal bovine chondrocytes and osteoarthritic human chondrocytes cultures [31,32]. The same authors described a trended reduction of pain in an OA model when dogs were fed with the corresponding mixture for 3 months [33]. Remarkably, a synergistic effect greater than the sum of the effects observed separately for each compound has even been described for fisetin and docosahexaenoic acid (DHA) on bone with an inhibition of osteoclastogenesis when compounds were mixed [34,35]. OPCO gathers part of the OPC and PHO compositions regarding HT and PCy content. Thus, we wondered if OPCO composition may result in synergistic or cumulative benefits. Unfortunately, we found no synergy but, somehow, found a few cumulative effects that depend on the target. Actually, additional effects were observed for NO and PGE2 production in vitro in HAC but not for MMP13. This observation suggests that, in contrast to MMP13, PHO (HT) and OPC (PCy) extracts may influence NO and PGE2 production through different pathways. Both PHO (HT) and OPC (PCy) almost completely blocked p65 translocation, thus providing insight regarding the absence of a cumulative effect of the OPCO (HT/PCy) on the NF-κB pathway. Thus, while NF-κB blocking by PHO (HT) or OPC (PCy) may at least partly explain the absence of a cumulative effect by OPCO (HT/PCy) on MMP13, it does not fully address the mechanism for cumulative effect on PGE2 and NO, supporting the involvement of other signaling pathways.

To determine the potential benefit of PHO and OPC extract on joints, we used a relevant cell model of human articular chondrocytes. However, “in a real life”, cells never “see” an extract or any native molecules. Cells rather “deal with” metabolites that originate from food processing, digestion, absorption, and transformation through the digestive track. Therefore, to improve the relevance of our data in humans, we designed an original clinical approach based on human serum enrichment.

We previously published the effect of both HT and PCy in a rabbit model of OA. According to the protocol, the quantity of polyphenols (including HT and PCy) delivered at once and given by force-feeding was set to 100 mg/kg of body weight [23,24]. According to metabolic weight calculation, this dose corresponds to 45 mg of polyphenols per kilogram of body weight in humans or a single dose of 3 g of polyphenols. Thus, volunteers were given orally 3.2 g of the OPCO extract containing 95% of polyphenols including 15% of PCy and 6% of HT. The recommendation for the polyphenol daily intake surrounds 1 g [36]. Although slightly higher, the dose delivered remains in a nutritional range and, overall, ten times lower than the dose delivered to our mouse model of OA showing positive impact of the extract on the AO score with no side effects [23,24].

According to our patent (DIRV#180058 INRA) [22], the human serum did not alter cell growth and proliferation rate was found similar between FCS and human serum (+31% and +35% between 24 and 48 h, respectively). Moreover, enrichment had no influence on cell growth, allowing strict comparison for biological effect. IL-1 β induction of NO, PGE2, and MMP13 was quite similar independently of the type of serum (FCS or human), confirming the physiological relevance of both the model and the approach (+159% and +121% for NO; +3982% and +2697% for PGE2; and +296% and +211% for MMP13, in the presence of FCS or human serum, respectively). Slight variations were observed depending on HAC batch. Indeed, differences between Figure 2 and Figure 5 were related to donors but, beside the seemingly discrepancy observed, variations remained in the same range (+159% and +500% for NO; +3982% and +4800% for PGE2; and +296% and +427% for MMP13 depending on HAC batches).

**Figure A1:**
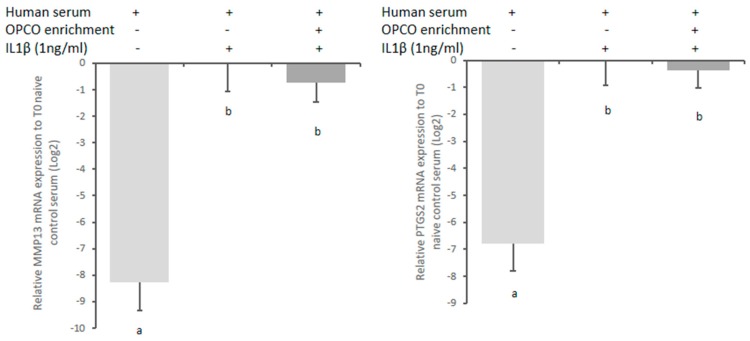
Effect of human serum enriched with OPCO metabolites on MMP-13 and PTGS2 mRNA expression levels in primary human articular chondrocytes: Human articular chondrocytes were preincubated with human serum enriched with OPCO metabolites for 24 h and stimulated with IL-1 β (1 ng/mL) for additional 24 h (- absence; + presence). MMP-13 and PTGS2 mRNA expression levels in primary human articular chondrocytes was measured by RT-PCR (PowerUp SYBRgreen, Applied Biosystems). β-Actine was used as a housekeeping gene. Primers were designed as follows: PTGS2-F: CTT CAC GCA TCA GTT TTT CAA G; PTGS2-R: TCA CCG TAA ATA TGA TTT AAG TCC AC; MMP13-F: CCA GTC TCC GAG GAG AAA CA; MMP13-R: AAA AAC AGC TCC GCA TCA AC; ACTB-F: ATT GGC AAT GAG CGG TTC; and ACTB-R: GGA TGC CAC AGG ACT CCA. The incubation with IL-1 β (1 ng/mL) was set as the control condition to enlighten the effect of the human serum enriched with OPCO metabolites. Despite a trend, there was no significant impact of the enrichment. Groups with significant differences (*P* < 0.05) are indicated with different letters (a and b).

Although, it had slight but no significant effect on transcripts (Figure A1), the serum enrichment in OPCO extract metabolites significantly limited the IL-1 β-induction of those three markers (−8.1% for NO, −50.7% for PGE2, and −5.8% for MMP13 production). These data further confirm at a clinical level the chondroprotective role of the extract that we observed in vitro upon pro-inflammatory stress. Moreover, these ex vivo results perfectly match with literature data from other clinical trials regarding anti-inflammatory and antioxidant capabilities of “polyphenol strategies”. To date, a reconstituted freeze-dried strawberry beverage (50 g/day) given to obese adults with radiographic evidence of knee OA significantly lowered serum biomarkers of inflammation including (IL)-6, IL-1 β, and cartilage degradation mediators including matrix metalloproteinase (MMP)-3 [37]. Forty g of freeze-dried blueberry powder given daily for four months to adults with symptomatic knee OA significantly decreased (WOMAC, Western Ontario and McMaster Universities Osteoarthritis Index) total score and subgroups, including pain, stiffness, and difficulty to perform daily activities [38]. Pomegranate juice decreases serum levels of matrix metalloproteinase (MMP)-13 and improves antioxidant status (glutathione peroxidase) in patients with knee OA [39]. Remarkably, pomegranates and blueberries are well-acknowledged sources of both PCy and anthocyanins [40,41]. Curcuminoids in combination with hydroxytyrosol attenuates systemic oxidative stress in patients with mild-to-moderate primary knee osteoarthritis when given at the dose of 1500 mg/day for a period of 6 weeks [42].

Among other classes of polyphenols, resveratrol has attracted much attention. Used as an adjuvant with meloxicam, resveratrol (500 mg/day) significantly improves pain, knee functions, and WOMAC scores with no side effects in patients with knee osteoarthritis [43]. Given twice daily at the dose of 75 mg for 14 weeks, it reduces chronic pain in healthy postmenopausal women with age-related osteoarthritis [44]. Finally, in patients with rheumatoid arthritis, activity score assessment for 28 joints was significantly lowered when their conventional treatment was supplemented with a daily capsule of 1 g of resveratrol for 3 months [45].

Although minor compared to the in vitro observations, the significant limitation of NO, PGE2, and MMP13 release reported by our ex vivo methodology strongly supports the relevance of a “realistic” polyphenol strategy for the management of chronic OA. The rationale of our pioneering clinical methodology was recently reinforced as polyphenol metabolites were found to distribute into the serum but, more interestingly, into the synovial fluid of patients with osteoarthritis [46]. Remarkably, our conclusions strictly parallel with clinical trials that were run for several months and needed to recruit hundreds of volunteers to evidence the chondroprotective effect and to reach a statistical significance. In this context, although our approach would not substitute a “regular” clinical trial, this innovative clinical approach may have helped the authors to rapidly explore human data before moving into a huge clinical investment [22].

## 5. Conclusions

From a global perspective, our results correlate not only with literature data on the positive role of polyphenols but also, most notably, with those from olive and grape origin on cartilage. Using a pioneering clinical screening approach, we further support the articular sparing ability of HT- and PCy-enriched extracts and the importance of the NF-κB pathway on the mode of action of polyphenols on cartilage metabolism. Accordingly, grapeseed and olive extracts characterized for their HT and PCy content stand as a relevant nutritional opportunity for advanced strategies to manage osteoarticular health conditions.

## 6. Patents

The human ex vivo methodology used in this study has been registered as a written invention disclosure by the French National Institute for Agronomic Research (INRA) (DIRV#18-0058). Clinic’n’Cell^®^ has been registered as a mark [22].
